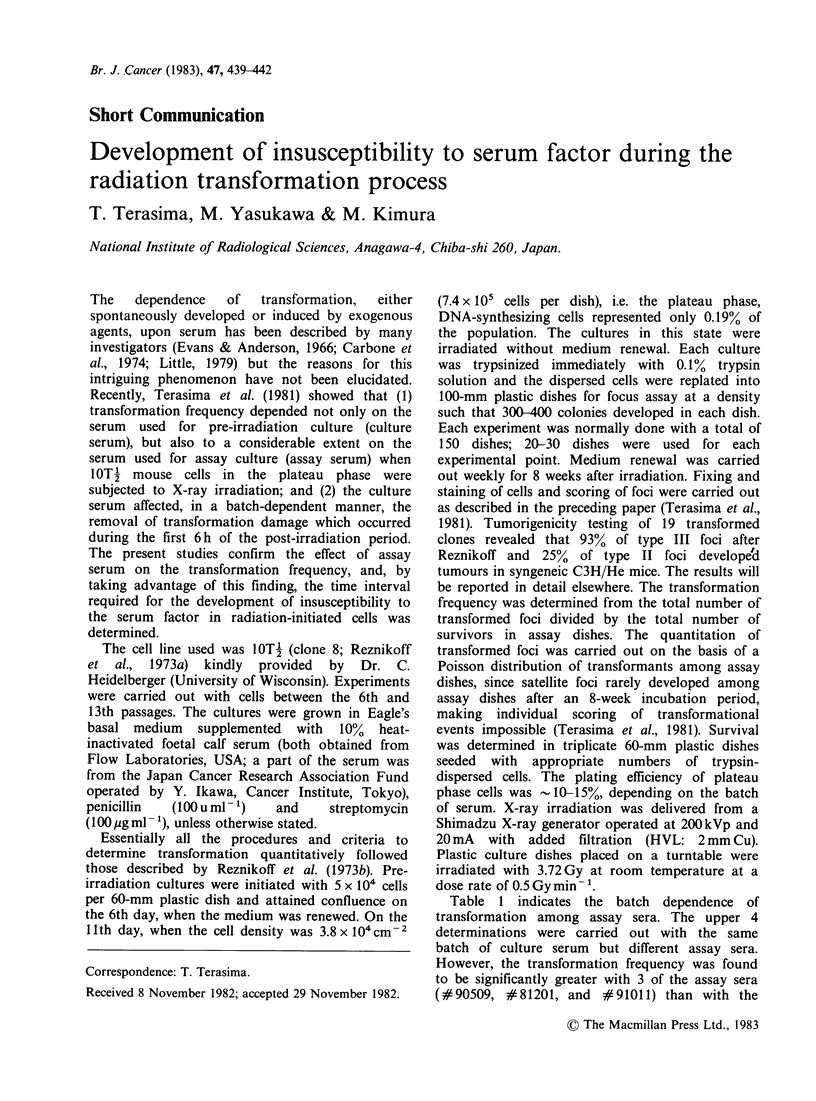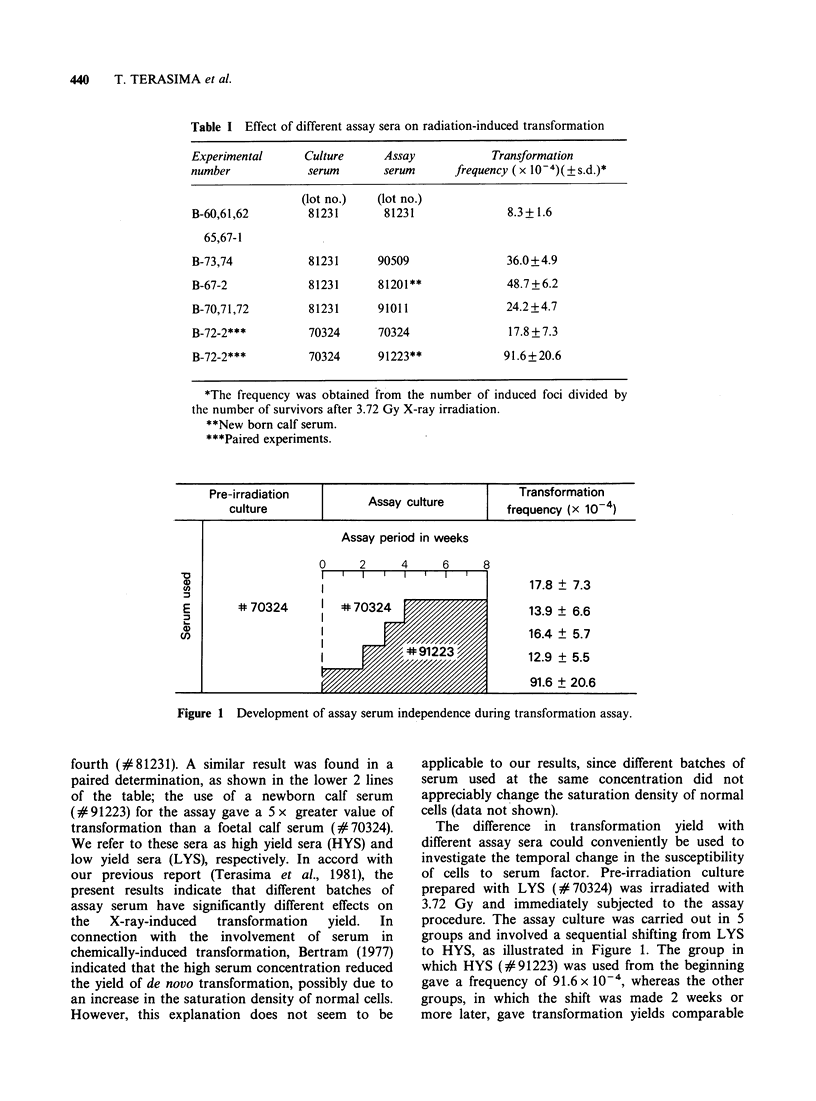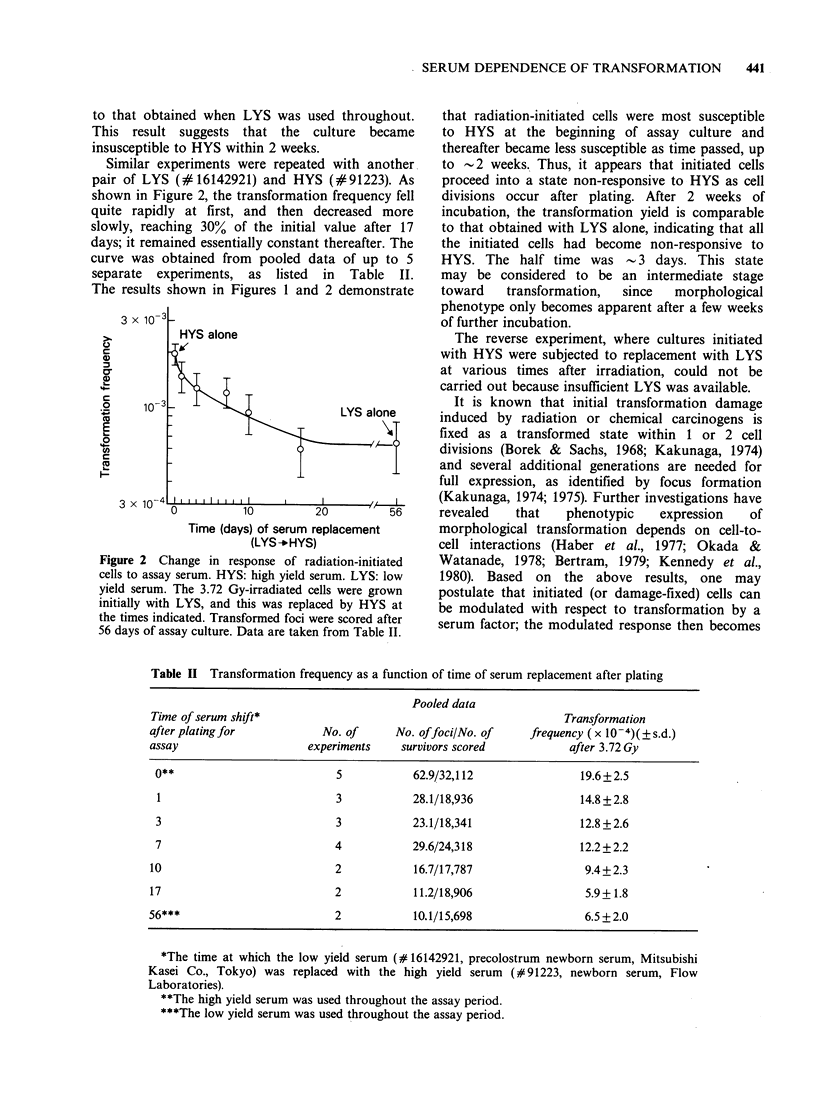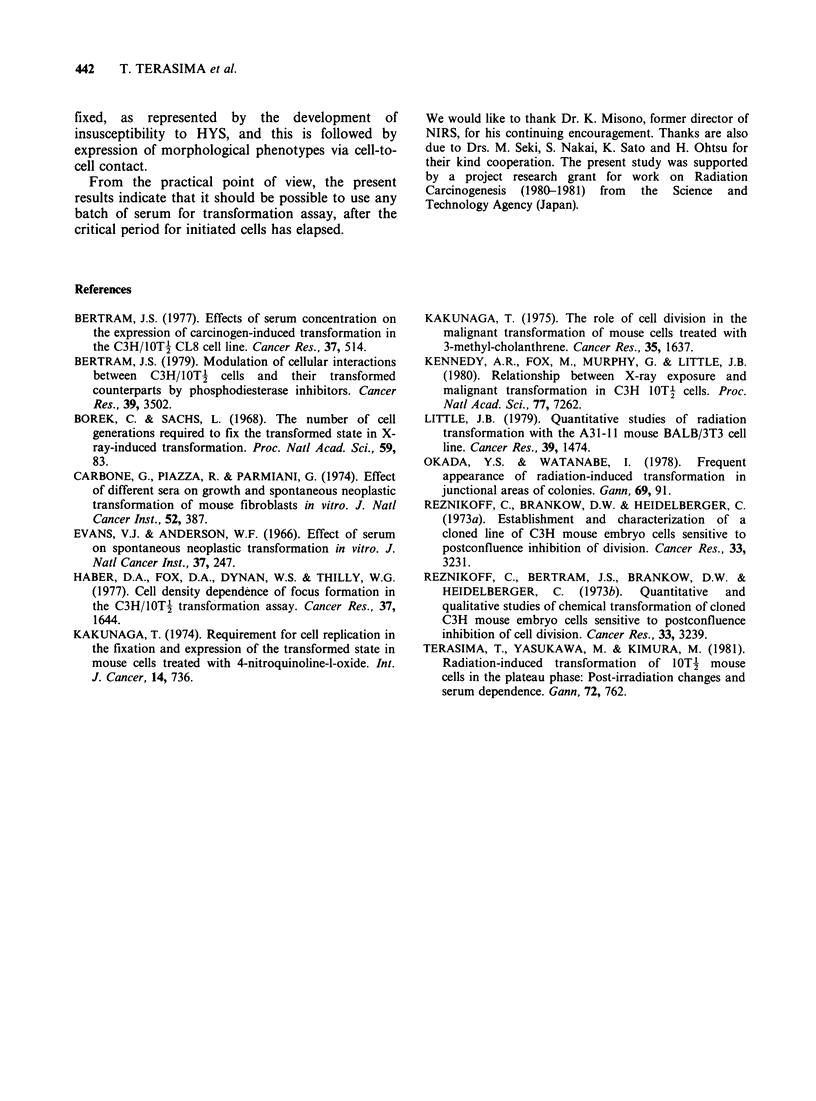# Development of insusceptibility to serum factor during the radiation transformation process.

**DOI:** 10.1038/bjc.1983.67

**Published:** 1983-03

**Authors:** T. Terasima, M. Yasukawa, M. Kimura


Br. J. Cancer (1983), 47, 439–442

### Short Communication

### Development of insusceptibility to serum factor during the radiation transformation process

T. Terasima, M. Yasukawa & M. Kimura

*National Institute of Radiological Sciences, Anagawa-4, Chiba-shi 260, Japan.*

The dependence of transformation, either spontaneously developed or induced by exogenous agents, upon serum has been described by many investigators (Evans & Anderson, 1966; Carbone *et al.*, 1974; Little, 1979) but the reasons for this intriguing phenomenon have not been elucidated. Recently, Terasima *et al.* (1981) showed that (1) transformation frequency depended not only on the serum used for pre-irradiation culture (culture serum), but also to a considerable extent on the serum used for assay culture (assay serum) when $10T\frac{1}{2}$ mouse cells in the plateau phase were subjected to X-ray irradiation; and (2) the culture serum affected, in a batch-dependent manner, the removal of transformation damage which occurred during the first 6 h of the post-irradiation period. The present studies confirm the effect of assay serum on the transformation frequency, and, by taking advantage of this finding, the time interval required for the development of insusceptibility to the serum factor in radiation-initiated cells was determined.

The cell line used was $10T\frac{1}{2}$ (clone 8; Reznikoff *et al.*, 1973*a*) kindly provided by Dr. C. Heidelberger (University of Wisconsin). Experiments were carried out with cells between the 6th and 13th passages. The cultures were grown in Eagle's basal medium supplemented with 10% heat-inactivated foetal calf serum (both obtained from Flow Laboratories, USA; a part of the serum was from the Japan Cancer Research Association Fund operated by Y. Ikawa, Cancer Institute, Tokyo), penicillin ($100\,u\,ml^{-1}$) and streptomycin ($100\,\mu g\,ml^{-1}$), unless otherwise stated.

Essentially all the procedures and criteria to determine transformation quantitatively followed those described by Reznikoff *et al.* (1973*b*). Pre-irradiation cultures were initiated with $5\times10^4$ cells per 60-mm plastic dish and attained confluence on the 6th day, when the medium was renewed. On the 11th day, when the cell density was $3.8\times10^4\,cm^{-2}$ ($7.4\times10^5$ cells per dish), i.e. the plateau phase, DNA-synthesizing cells represented only 0.19% of the population. The cultures in this state were irradiated without medium renewal. Each culture was trypsinized immediately with 0.1% trypsin solution and the dispersed cells were replated into 100-mm plastic dishes for focus assay at a density such that 300–400 colonies developed in each dish. Each experiment was normally done with a total of 150 dishes; 20–30 dishes were used for each experimental point. Medium renewal was carried out weekly for 8 weeks after irradiation. Fixing and staining of cells and scoring of foci were carried out as described in the preceding paper (Terasima *et al.*, 1981). Tumorigenicity testing of 19 transformed clones revealed that 93% of type III foci after Reznikoff and 25% of type II foci developed tumours in syngeneic C3H/He mice. The results will be reported in detail elsewhere. The transformation frequency was determined from the total number of transformed foci divided by the total number of survivors in assay dishes. The quantitation of transformed foci was carried out on the basis of a Poisson distribution of transformants among assay dishes, since satellite foci rarely developed among assay dishes after an 8-week incubation period, making individual scoring of transformational events impossible (Terasima *et al.*, 1981). Survival was determined in triplicate 60-mm plastic dishes seeded with appropriate numbers of trypsin-dispersed cells. The plating efficiency of plateau phase cells was ~10–15%, depending on the batch of serum. X-ray irradiation was delivered from a Shimadzu X-ray generator operated at 200 kVp and 20 mA with added filtration (HVL: 2 mm Cu). Plastic culture dishes placed on a turntable were irradiated with 3.72 Gy at room temperature at a dose rate of $0.5\,Gy\,min^{-1}$.

Table 1 indicates the batch dependence of transformation among assay sera. The upper 4 determinations were carried out with the same batch of culture serum but different assay sera. However, the transformation frequency was found to be significantly greater with 3 of the assay sera (#90509, #81201, and #91011) than with the

---

Correspondence: T. Terasima.

Received 8 November 1982; accepted 29 November 1982.

© The Macmillan Press Ltd., 1983

**Table I** Effect of different assay sera on radiation-induced transformation

| *Experimental number* | *Culture serum* (lot no.) | *Assay serum* (lot no.) | *Transformation frequency* ($\times 10^{-4}$)($\pm$s.d.)* |
|---|---|---|---|
| B-60,61,62 65,67-1 | 81231 | 81231 | $8.3 \pm 1.6$ |
| B-73,74 | 81231 | 90509 | $36.0 \pm 4.9$ |
| B-67-2 | 81231 | 81201** | $48.7 \pm 6.2$ |
| B-70,71,72 | 81231 | 91011 | $24.2 \pm 4.7$ |
| B-72-2*** | 70324 | 70324 | $17.8 \pm 7.3$ |
| B-72-2*** | 70324 | 91223** | $91.6 \pm 20.6$ |

*The frequency was obtained from the number of induced foci divided by the number of survivors after 3.72 Gy X-ray irradiation.
**New born calf serum.
***Paired experiments.

**Figure 1** Development of assay serum independence during transformation assay.

fourth (#81231). A similar result was found in a paired determination, as shown in the lower 2 lines of the table; the use of a newborn calf serum (#91223) for the assay gave a $5\times$ greater value of transformation than a foetal calf serum (#70324). We refer to these sera as high yield sera (HYS) and low yield sera (LYS), respectively. In accord with our previous report (Terasima *et al.*, 1981), the present results indicate that different batches of assay serum have significantly different effects on the X-ray-induced transformation yield. In connection with the involvement of serum in chemically-induced transformation, Bertram (1977) indicated that the high serum concentration reduced the yield of *de novo* transformation, possibly due to an increase in the saturation density of normal cells. However, this explanation does not seem to be applicable to our results, since different batches of serum used at the same concentration did not appreciably change the saturation density of normal cells (data not shown).

The difference in transformation yield with different assay sera could conveniently be used to investigate the temporal change in the susceptibility of cells to serum factor. Pre-irradiation culture prepared with LYS (#70324) was irradiated with 3.72 Gy and immediately subjected to the assay procedure. The assay culture was carried out in 5 groups and involved a sequential shifting from LYS to HYS, as illustrated in Figure 1. The group in which HYS (#91223) was used from the beginning gave a frequency of $91.6 \times 10^{-4}$, whereas the other groups, in which the shift was made 2 weeks or more later, gave transformation yields comparable to that obtained when LYS was used throughout. This result suggests that the culture became insusceptible to HYS within 2 weeks.

Similar experiments were repeated with another pair of LYS (#16142921) and HYS (#91223). As shown in Figure 2, the transformation frequency fell quite rapidly at first, and then decreased more slowly, reaching 30% of the initial value after 17 days; it remained essentially constant thereafter. The curve was obtained from pooled data of up to 5 separate experiments, as listed in Table II. The results shown in Figures 1 and 2 demonstrate

**Figure 2** Change in response of radiation-initiated cells to assay serum. HYS: high yield serum. LYS: low yield serum. The 3.72 Gy-irradiated cells were grown initially with LYS, and this was replaced by HYS at the times indicated. Transformed foci were scored after 56 days of assay culture. Data are taken from Table II.

that radiation-initiated cells were most susceptible to HYS at the beginning of assay culture and thereafter became less susceptible as time passed, up to ~2 weeks. Thus, it appears that initiated cells proceed into a state non-responsive to HYS as cell divisions occur after plating. After 2 weeks of incubation, the transformation yield is comparable to that obtained with LYS alone, indicating that all the initiated cells had become non-responsive to HYS. The half time was ~3 days. This state may be considered to be an intermediate stage toward transformation, since morphological phenotype only becomes apparent after a few weeks of further incubation.

The reverse experiment, where cultures initiated with HYS were subjected to replacement with LYS at various times after irradiation, could not be carried out because insufficient LYS was available.

It is known that initial transformation damage induced by radiation or chemical carcinogens is fixed as a transformed state within 1 or 2 cell divisions (Borek & Sachs, 1968; Kakunaga, 1974) and several additional generations are needed for full expression, as identified by focus formation (Kakunaga, 1974; 1975). Further investigations have revealed that phenotypic expression of morphological transformation depends on cell-to-cell interactions (Haber *et al.*, 1977; Okada & Watanade, 1978; Bertram, 1979; Kennedy *et al.*, 1980). Based on the above results, one may postulate that initiated (or damage-fixed) cells can be modulated with respect to transformation by a serum factor; the modulated response then becomes

**Table II** Transformation frequency as a function of time of serum replacement after plating

| *Time of serum shift* after plating for assay* | *Pooled data* No. of experiments | *No. of foci/No. of survivors scored* | *Transformation frequency* ($\times 10^{-4}$)($\pm$s.d.) *after* 3.72 *Gy* |
|---|---|---|---|
| 0** | 5 | 62.9/32,112 | 19.6±2.5 |
| 1 | 3 | 28.1/18,936 | 14.8±2.8 |
| 3 | 3 | 23.1/18,341 | 12.8±2.6 |
| 7 | 4 | 29.6/24,318 | 12.2±2.2 |
| 10 | 2 | 16.7/17,787 | 9.4±2.3 |
| 17 | 2 | 11.2/18,906 | 5.9±1.8 |
| 56*** | 2 | 10.1/15,698 | 6.5±2.0 |

*The time at which the low yield serum (#16142921, precolostrum newborn serum, Mitsubishi Kasei Co., Tokyo) was replaced with the high yield serum (#91223, newborn serum, Flow Laboratories).

**The high yield serum was used throughout the assay period.

***The low yield serum was used throughout the assay period.

fixed, as represented by the development of insusceptibility to HYS, and this is followed by expression of morphological phenotypes via cell-to-cell contact.

From the practical point of view, the present results indicate that it should be possible to use any batch of serum for transformation assay, after the critical period for initiated cells has elapsed.

We would like to thank Dr. K. Misono, former director of NIRS, for his continuing encouragement. Thanks are also due to Drs. M. Seki, S. Nakai, K. Sato and H. Ohtsu for their kind cooperation. The present study was supported by a project research grant for work on Radiation Carcinogenesis (1980–1981) from the Science and Technology Agency (Japan).

**References**

BERTRAM, J.S. (1977). Effects of serum concentration on the expression of carcinogen-induced transformation in the C3H/10T$\frac{1}{2}$ CL8 cell line. *Cancer Res.*, **37**, 514.

BERTRAM, J.S. (1979). Modulation of cellular interactions between C3H/10T$\frac{1}{2}$ cells and their transformed counterparts by phosphodiesterase inhibitors. *Cancer Res.*, **39**, 3502.

BOREK, C. & SACHS, L. (1968). The number of cell generations required to fix the transformed state in X-ray-induced transformation. *Proc. Natl Acad. Sci.*, **59**, 83.

CARBONE, G., PIAZZA, R. & PARMIANI, G. (1974). Effect of different sera on growth and spontaneous neoplastic transformation of mouse fibroblasts *in vitro*. *J. Natl Cancer Inst.*, **52**, 387.

EVANS, V.J. & ANDERSON, W.F. (1966). Effect of serum on spontaneous neoplastic transformation *in vitro*. *J. Natl Cancer Inst.*, **37**, 247.

HABER, D.A., FOX, D.A., DYNAN, W.S. & THILLY, W.G. (1977). Cell density dependence of focus formation in the C3H/10T$\frac{1}{2}$ transformation assay. *Cancer Res.*, **37**, 1644.

KAKUNAGA, T. (1974). Requirement for cell replication in the fixation and expression of the transformed state in mouse cells treated with 4-nitroquinoline-l-oxide. *Int. J. Cancer*, **14**, 736.

KAKUNAGA, T. (1975). The role of cell division in the malignant transformation of mouse cells treated with 3-methyl-cholanthrene. *Cancer Res.*, **35**, 1637.

KENNEDY, A.R., FOX, M., MURPHY, G. & LITTLE, J.B. (1980). Relationship between X-ray exposure and malignant transformation in C3H 10T$\frac{1}{2}$ cells. *Proc. Natl Acad. Sci.*, **77**, 7262.

LITTLE, J.B. (1979). Quantitative studies of radiation transformation with the A31-11 mouse BALB/3T3 cell line. *Cancer Res.*, **39**, 1474.

OKADA, Y.S. & WATANABE, I. (1978). Frequent appearance of radiation-induced transformation in junctional areas of colonies. *Gann*, **69**, 91.

REZNIKOFF, C., BRANKOW, D.W. & HEIDELBERGER, C. (1973*a*). Establishment and characterization of a cloned line of C3H mouse embryo cells sensitive to postconfluence inhibition of division. *Cancer Res.*, **33**, 3231.

REZNIKOFF, C., BERTRAM, J.S., BRANKOW, D.W. & HEIDELBERGER, C. (1973*b*). Quantitative and qualitative studies of chemical transformation of cloned C3H mouse embryo cells sensitive to postconfluence inhibition of cell division. *Cancer Res.*, **33**, 3239.

TERASIMA, T., YASUKAWA, M. & KIMURA, M. (1981). Radiation-induced transformation of 10T$\frac{1}{2}$ mouse cells in the plateau phase: Post-irradiation changes and serum dependence. *Gann*, **72**, 762.